# Knowledge, Attitudes, and Practices of Dental Students from Romania Regarding Self-Perceived Risk and Prevention of Infectious Diseases

**DOI:** 10.3390/dj12040097

**Published:** 2024-04-10

**Authors:** Florentina Iuliana Dincă, Bogdan-Alexandru Dimitriu, Oana Săndulescu, Valentin Daniel Sîrbu, Mihai Săndulescu

**Affiliations:** 1 PhD Candidate, Doctoral School, Carol Davila University of Medicine and Pharmacy, 37 Dionisie Lupu Street, 020022 Bucharest, Romania; 2 Department of Endodontics, Faculty of Dentistry, Carol Davila University of Medicine and Pharmacy, 17-23 Calea Plevnei, 020022 Bucharest, Romania; 3 Department of Infectious Diseases I, Faculty of Medicine, Carol Davila University of Medicine and Pharmacy, 8 Eroii Sanitari Boulevard, 050474 Bucharest, Romania; 4 National Institute for Infectious Diseases “Prof. Dr. Matei Balș”, No. 1 Dr. Calistrat Grozovici Street, 021105 Bucharest, Romania; 5 Department of Implant-Prosthetic Therapy, Faculty of Dentistry, Carol Davila University of Medicine and Pharmacy, 17-23 Calea Plevnei, 020022 Bucharest, Romaniamihai.sandulescu@umfcd.ro (M.S.)

**Keywords:** awareness, bloodborne pathogens, vaccine-preventable diseases, dental student, risk, perception, attitude of health personnel

## Abstract

University education is a leading source of information for dental practitioners. Particular emphasis should be given to determining the extent to which students acquire positive knowledge, attitudes, and practices (KAP) and positive metacompetences beyond the scope of each studied dental discipline. We performed a cross-sectional questionnaire-based study among dentistry students from Romania to assess self-perceived risk of infectious diseases and their KAP on topics related to infectious disease prevention. The surveyed students presented good knowledge regarding personal protective equipment (PPE), and their PPE practices significantly correlated with the perceived usefulness of PPE. Only 45.1% correctly recognized all vaccine-preventable diseases (VPDs), but knowledge regarding VPDs significantly improved with increasing year of study (τ_b_ = 0.298, *p* = 0.001), confirming a positive education effect. Awareness regarding the need for screening for bloodborne viruses is poor; the majority of students had never performed a test for hepatitis C virus infection (HCV) (59.4%) or for human immunodeficiency virus (HIV) infection (60.4%). Furthermore, most respondents incorrectly considered themselves at high or very high risk of acquiring BBV, and perceived risk was inversely correlated with willingness to treat patients with hepatitis B virus (HBV) infection (τ_b_ = −0.214, *p* = 0.018), HCV infection (τ_b_ = −0.234, *p* = 0.013), or HIV infection (τ_b_ = −0.242, *p* = 0.006). This led to 3.0% of respondents stating that they would hypothetically deny dental treatment to a patient with HBV infection, 5.0% for HCV infection, and 10.9% for HIV infection, the proportion being significantly higher for HIV (z = −2.2, *p* = 0.026). In conclusion, better knowledge is needed among dental students regarding their own vaccination history, screening for bloodborne viruses, accurate estimates for their risk of acquiring bloodborne viruses during routine dental practice, and the existence of post-exposure measures following occupational exposure. Improving student knowledge and awareness could translate into a higher willingness to treat patients with chronic viral infections and into a safer and more inclusive dental practice. We propose an adaptation to the university curriculum to cover these key areas for targeted focus to empower future dental practitioners and to facilitate the improvement of across-discipline metacompetences for infection prevention and control.

## 1. Introduction

University education is among the most trusted sources of information for dental practitioners worldwide [1,2,3,4]. For this reason, particular emphasis should be put on determining the extent to which undergraduate students acquire not only the information they need regarding dental practice but also positive knowledge, attitudes and practices (KAP) and positive metacompetences beyond the scope of each of the studied dental specialty topics.

Dentists represent a key subset of healthcare workers and are exposed to particular professional risks through the nature of their work. The biological safety of the medical act, for both patient and practitioner, should be safeguarded for all healthcare professional categories. For this reason, setting-specific infection prevention and control (IPC) norms and regulations are in place and are complemented by recommendations and training to follow the standard precautions, to correctly use personal protective equipment (PPE), and to assess and manage biological risk by ensuring pre-exposure prophylaxis through vaccination as well as immediate access to post-exposure interventions to mitigate risk in case of accidental exposure. In order for all these recommendations to function as intended and to ensure the safety of the medical act, they should be known, understood, and consistently implemented into dental practice.

According to the current curriculum, students in our university learn about IPC for dentistry practice in their third year of undergraduate study and then go on to study infectious diseases and epidemiology in their fourth year of dental school, for a total combined amount of 42 theoretical and 42 practical hours. However, the degree to which students integrate the information acquired during undergraduate training into their future clinical practice remains to be determined, as much emphasis is currently put on the need to improve students’ preparedness for future independent practice [5].

Through the current study, we aimed to assess dental students’ self-perceived risk of infectious diseases and their KAP on topics related to the prevention of infectious diseases in order to explore and better understand key areas of targeted focus for future improvement of across-discipline IPC metacompetences.

## 2. Materials and Methods

### 2.1. Study Design

This was a cross-sectional questionnaire-based study among dentistry students from Romania, designed to assess their knowledge, attitudes, and practices (KAP) regarding the main measures that they take in routine dental practice to protect themselves from infectious diseases. 

### 2.2. Study Questionnaire

A questionnaire with 5 sections was constructed for the purpose of the current study to assess different domains related to infection prevention in dentistry. The first section included a description of the study and the respondent’s option to consent or to withdraw from completing the questionnaire. The second section collected demographic information regarding the respondent and evaluated the awareness and the prior training received regarding PPE, as well as the students’ attitudes and practices related to hand hygiene and PPE use. The following types of PPE were specifically assessed: surgical mask, filtering facepiece (FFP), non-sterile gloves, sterile surgical gloves, protection goggles, face shield, surgical cap, and single-use gown. The third section evaluated student KAP regarding chronic viral infections such as hepatitis B virus (HBV), hepatitis C virus (HCV), or human immunodeficiency virus (HIV) infection; the questions assessed the students’ previous experience in working with patients who had chronic viral infections, their self-perceived risk of acquiring infection at work, and their willingness to treat this category of patients. The fourth section reviewed the respondents’ history of accidental occupational exposure and, if applicable, of accessing post-exposure prophylaxis and testing the source patient. The fifth and last section of the questionnaire addressed the students’ self-reported vaccination status, as well as their KAP regarding vaccine-preventable diseases (VPDs) and history of undergoing screening for bloodborne viruses (BBV).

The draft questionnaire was initially piloted in a group of 10 dentistry students and question phrasing was adapted for clarity in a few instances. No questions were removed or replaced during the piloting process. These 10 pilot responses were not included in the final analysis.

The link to the online questionnaire was distributed to dental student associations from three main university centers throughout the country: Bucharest, Iași, and Craiova. It was addressed to students from any university year (1 through 6) and the questionnaire remained open for a duration of 4 weeks, from 27 October 2023 to 24 November 2023. The questionnaire was self-administered, participation in the study was voluntary, anonymous, and without compensation.

### 2.3. Statistical Analysis

We present descriptive statistics by reporting the absolute and relative frequencies for categorical or ordinal variables. For the statistical analysis, we applied the Z score test in order to determine the association between two population proportions and Kendall’s Tau-b to determine the correlation between two ordinal variables; *p* values < 0.05 were considered statistically significant. Statistical analysis was performed with SPSS Statistics (version 25, IBM Corp., Armonk, NY, USA).

### 2.4. Ethics Approval

Ethics approval for the study was granted by the Bioethics Committee of the National Institute for Infectious Diseases “Prof. Dr. Matei Balș”, Bucharest, Romania.

## 3. Results

A total number of 102 students accepted the invitation to complete the survey. One of the respondents did not provide consent, leaving a total number of 101 valid answers. Most respondents (90.1%) were female and most (*n* = 91, 90.1%) were studying in Bucharest, with the universities from Iași and Craiova accounting for five students each. The age range was 19 to 32 years, with a median of 23 (IQR: 21–24) years. Almost half (44.6%) of the respondents were enrolled in their 6th year of study, followed by 3rd year students (22.8%) and all other study years, with varying frequencies (Table 1). The majority of them (79.2%) were also involved in some type of clinical activity. 

### 3.1. Awareness and Prior Training Regarding Personal Protective Equipment

We first assessed the students’ self-reported prior training regarding PPE wear. Most students reported having received information regarding the correct use for all types of PPE surveyed: surgical mask (97%), FFP (84.2%), non-sterile gloves (97%), sterile surgical gloves (91.1%), protection goggles (98%), face shield (93.1%), surgical cap (88.1%), and single-use gown (92.1%).

University ranked highest among the sources of information cited (84.2%), followed closely by the clinical practice setting (81.2%) and decreasing frequencies for other sources, such as discussions with peers (64.2%), websites (37.6%), national public health authorities (35.6%), international authorities (19.8%), and online forums (12.9%). This question allowed the students to choose as many answers as needed; approximately one quarter of respondents chose two (25.7%), three (22.8%), and four (22.8%) information sources.

### 3.2. Attitudes and Practices Related to Personal Protective Equipment Use

All respondents stated that they had access to enough gloves in their clinical practice; however, 23.8% of them also stated that sometimes they had to buy non-sterile gloves themselves, 20.8% had to buy masks, and 3% had to buy sterile surgical gloves at some point in their practice. While only 3% of respondents stated that they did not have access to safety goggles in clinical practice, most of them (49.5%) stated that they wore them rarely or not at all. However, 82.2% of them correctly recognized the indication to wear goggles in clinical scenarios that carried a risk of splashing of biological fluids such as saliva or blood. 

While most (91.1%) respondents stated that they washed their hands before coming into contact with each patient, only 83.2% of them reported that they also washed their hands after taking off gloves, and 14.9% of them washed their gloves while wearing them.

**Figure 1:**
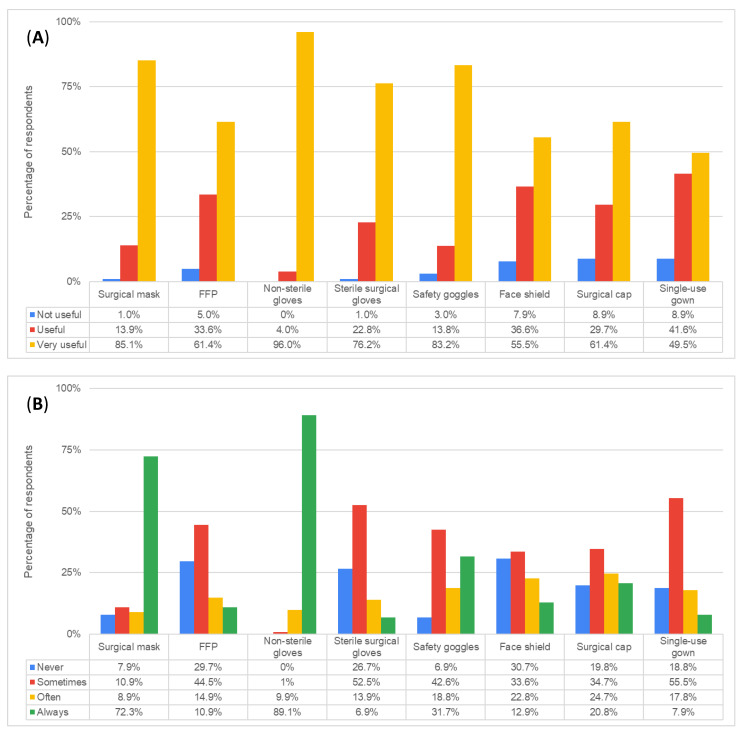
(**A**) Perceived usefulness of personal protective equipment by dental students. (**B**) Self-reported frequency of personal protective equipment by dental students. FFP, filtering facepiece.

Participants were asked to grade, in their opinion, the usefulness of each type of PPE for their practice. Non-sterile gloves ranked highest, followed by safety goggles, surgical masks, and sterile surgical gloves, all being considered “very useful” by more than three quarters of the respondents (Figure 1A).

We identified a positive correlation between the perceived usefulness and the reported use (Figure 1B) for almost all types of analyzed PPE: surgical masks (τ_b_ = 0.288, *p* = 0.002), FFP (τ_b_ = 0.320, *p* < 0.001), non-sterile gloves (τ_b_ = 0.273, *p* = 0.003), safety goggles (τ_b_ = 0.279, *p* = 0.002), face shields (τ_b_ = 0.500, *p* < 0.001), surgical caps (τ_b_ = 0.449, *p* < 0.001), and single-use gowns (τ_b_ = 0.328, *p* < 0.001).

### 3.3. Knowledge, Attitudes, and Practices Regarding Vaccine-Preventable Diseases

When asked to recognize vaccine-preventable diseases, 95% of respondents correctly identified HBV and 91.1% correctly identified HPV. However, a very large percentage of respondents incorrectly considered that HCV and HIV are vaccine-preventable, in 37.6% and 22.8% of cases, respectively. Only 45.1% of the students responded correctly to this question regarding VPDs, and there was a significant positive correlation between knowledge regarding VPDs and year of study (τ_b_ = 0.298, *p* = 0.001). 

When reporting their own vaccination status, 87.1% of participants stated that they were vaccinated against COVID-19, 69.3% against MMR, 44.6% against varicella, 28.7% against influenza (recently), and 22.8% against HPV. While 67.3% of the participants reported that they were vaccinated against diphtheria–tetanus–*pertussis*, only 17% of them had actually received a booster within the past 10 years, while 61% had not and 17% did not know.

Most respondents (84.2%) stated that they knew their vaccine status for HBV. Overall, 49.5% stated that they were vaccinated and 32.7% stated that they were not vaccinated. When asked whether they had ever checked their anti-HBs levels, 11% stated that they did not know, 60.4% stated that they never had, and a total of 27.8% stated that they had (of which 11.9% had done so in the current year, 14.9% within the past 2–5 years, and 1% more than 5 years ago). Among the twenty-eight who had previously determined their anti-HBs titer, only half remembered the approximate results: seven had levels < 10 mIU/mL, four had levels between 10–99 mIU/mL, and four had levels > 100 mIU/mL; two of the anti-HBs titers < 10 mIU/mL were reported by students who declared that they had not been previously vaccinated, and the rest of the values were reported by students who reported prior vaccination.

The large majority of the students stated that they had never performed an HCV test (59.4%) or an HIV test (60.4%), while 10.9% and 7.9%, respectively, did not know.

### 3.4. Knowledge, Attitudes, and Practices Regarding Chronic Viral Infections

A small proportion of respondents had previously performed dental procedures for a patient who had HBV infection (32.7%), HCV infection (22.8%), or HIV infection (11.9%). The self-perceived risk of infection was reported as high or very high by 66.3% of students for HBV, 64.4% for HCV, and 61.4% for HIV. 

When asked how they would react if a patient with one of these chronic viral infections approached them for dental treatment, an overall low proportion of respondents declared that they would deny them treatment if they had HBV infection (3.0%), but the rate increased numerically for HCV infection (5.0%) and statistically significantly for HIV infection (10.9%, z = −2.2, *p* = 0.026)—Table 2. 

The perceived risk was significantly inversely correlated with the attitude, for all types of chronic infections surveyed: HBV (τ_b_ = −0.214, *p* = 0.018), HCV (τ_b_ = −0.234, *p* = 0.013), and HIV (τ_b_ = −0.242, *p* = 0.006)—Table 2.

The year of study was not significantly correlated with perceived risk (*p* = 0.126, *p* = 0.157, *p* = 0.089) or willingness to perform dental procedures (*p* = 0.737, *p* = 0.893, *p* = 0.772).

In a subgroup analysis, among the 33 respondents who had previously worked with patients with HBV, none stated that they would refuse treatment in the future, one-third (*n* = 11) would choose to only perform procedures with minimal bleeding, and the other two-thirds (*n* = 22) stated that they would perform any type of procedure, including surgery. Interestingly, in this subgroup with previous experience, the proportion of those that perceived the risk as high or very high was lower (48.5%) than in the whole respondent group, but the difference was not statistically significant (z = 1.8, *p* = 0.067).

The same was true for the 23 respondents who had prior experience in working with patients with HCV infection: none would refuse future treatment, 39.1% would choose to perform procedures with minimal bleeding, and 60.9% would perform surgical procedures. In this subgroup, 52.2% perceived their risk as high and very high, compared to 64.4% in the overall group (z = 1.1, *p* = 0.276).

Among the 12 students who had prior experience in working with patients with HIV infection, the same pattern was seen: none would refuse treatment in the future, 33.3% would perform minimal-bleeding procedures, and 66.7% would perform surgical procedures. The risk was perceived as high and very high by 50% of respondents with prior experience, compared to 61.4% in the overall group (z = 0.8, *p* = 0.447).

When asked whether they would take supplementary precaution measures if they treated a patient with a chronic viral infection, only 2–3% of students stated that this is not necessary. Almost all respondents would take supplementary precautions for themselves (88–89%), for disinfecting and sterilizing the dental instruments (93–95%) and, to a lesser extent, for disinfecting the dental unit prior to the next patient (75%).

### 3.5. History of Accidental Occupational Exposure

**Figure 2:**
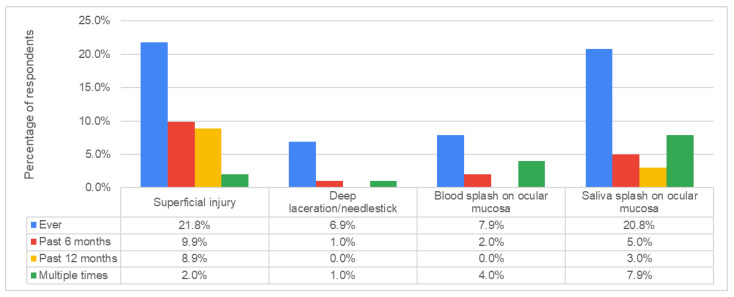
Self-reported routes and frequencies of accidental occupational exposure in dental students.

A total of 41 (40.6%) students reported to have had at least one prior episode of accidental occupational exposure, with the following frequencies of different types of exposures: superficial injury 21.8%, deep laceration or needlestick injury 6.9%, blood splash on ocular mucosa 7.9%, and saliva splash on ocular mucosa 20.8% (Figure 2).

When analyzing the subgroup of 41 respondents who reported a prior occupational exposure accident, 53.7% stated that this had occurred during rotary instrument use, 43.9% while preparing dental instruments for sterilization, 17.1% during endodontic instrument use, 9.8% during anesthesia, 7.3% during dental extraction, 2.4% during incision, 2.4% during orthodontic instrument use, and 22% during some other type of procedure. Most respondents chose one exposure route (56.1%) but almost one-third (29.3%) reported two exposure routes and 14.6% reported three prior exposure routes.

At the moment of exposure, only 55.0% of students investigated the source patient’s HBV, HCV, or HIV status. Following the accidental exposure, 80.5% of the respondents did not consult an infectious disease practitioner, 7.3% did not remember whether they did, and only 12.2% (*n* = 5) went in for a consultation. Of these five students, only two consulted an infectious disease practitioner in the first day following exposure, two went in for a consultation within the first 2–3 days, and one respondent went for a consultation more than one week after the exposure event. 

## 4. Discussion

In the current study, we found that most students referred to the university and their main dental practice clinic as their primary sources of information related to correct PPE use. Most survey respondents stated that they used masks and gloves consistently during their clinical practice; however, a worrisome finding was that 7.9% of students stated that they never wore masks, and less than half of respondents used safety goggles, despite confirming the availably of PPE in their clinical practice.

PPE use was significantly correlated with perceived usefulness, i.e., the more the students considered that a particular PPE item was useful in their clinical practice, the more likely they were to actually use that type of PPE, and this correlation was identified throughout the main PPE items assessed.

Hand hygiene is an essential component of standard precautions and is fundamental to infection prevention. Among the surveyed dental students, 91.1% correctly recognized one of the most important moments of hand hygiene, i.e., washing one’s hands before patient contact. However, a lower percentage (83.2%) also acknowledged the fact that hand hygiene should be performed after taking off gloves. This is an important issue, as it is one of the most frequently overlooked steps of hand hygiene [6]. While gloves are an important component of PPE, they are frequently misused in clinical practice, and their overuse has been associated with decreased compliance to hand hygiene [7]. Wearing gloves may give healthcare workers a sense of false security, and practitioners often fail to recognize that their hands do become contaminated under the gloves, and as a result, they fail to wash their hands when they take off the gloves [7].

Our results are in line with findings from a study that observed compliance to infection control practices in 3rd and 4th year dental students from the USA [6]; herein, mask use was observed in 97.9% of clinical encounters and preoperative handwashing in 88.3% of encounters [6]. However, the observed post-procedural handwashing in this cited study was much lower (26.4%) [6] than that self-reported in our study (83.2%).

When assessing knowledge regarding VPDs, we found that student knowledge positively correlated with increasing year of study, which is in line with data from other countries [8,9,10]. We also saw that most students correctly recognized VPDs such as HBV and HPV; however, there was a high degree of confusion between the types of viral hepatitis, with 37.6% of respondents considering HCV to be vaccine-preventable when in fact it is not. Furthermore, almost one quarter of the students, 22.8%, considered HIV infection to be vaccine-preventable, which is particularly worrisome because it reflects poor understanding and poor awareness regarding the different types of chronic viral infections, as can also be seen from the fact that most respondents considered themselves to be at high or very high risk of acquiring infection if they performed dental procedures to patients living with any of the three chronic infections assessed.

Interestingly, this self-perceived risk was higher among respondents who did not have any prior experience in working with patients with these chronic infections, and was also inversely correlated with attitudes, i.e., those who considered their risk to be higher were more likely to state that in the future they would hypothetically choose to avoid blood-prone procedures or deny treatment altogether to patients with chronic viral infections.

To this day, the stigma against people living with chronic viral infections such as HBV, HCV, or HIV, continues to represent a very important issue in many settings worldwide. For example, only 39.6% of the general population would report an accepting attitude towards patients living with HIV in Ethiopia in 2016, and acceptance was statistically associated with the level of knowledge regarding the infection [11]. A very recent study (2023) among medical sciences students from Iraq, including dentists, reported that 39.9% of the respondents would feel uncomfortable even sitting with a person with HBV infection, and 42.1% would feel uncomfortable shaking hands or hugging a person with HBV infection [12]; these findings could also be partly explained by an inaccurately high self-perception of risk and a lack of sufficient knowledge regarding the transmission routes; specifically, 79.1% of respondents considered healthcare workers to have higher risk than the general population of acquiring HBV infection, whereas 75.9% erroneously considered that HBV can be transmitted through shaking hands, coughing, sneezing, or through contaminated food or water [12]. This emphasizes the stringent need for more focused education regarding how BBV are and are not transmitted among medical science students.

In our current study, the rate of hypothetical treatment refusal was significantly higher for HIV compared to chronic liver infection such as HBV (10.9% vs. 3.0%). This suggests that one in ten patients with HIV could be denied dental treatment when approaching a random clinical practice, despite the fact that, even in the pre-universal antiretroviral therapy era, when many patients still had detectable viral loads, the risk of transmission was actually the lowest for HIV compared to other bloodborne viruses [13]. Unfortunately, this is in line with perceived stigma among people living with HIV, as shown from a recent report by the European Centre for Disease Prevention and Control (ECDC), where 23% of respondents from Europe and Central Asia stated that they had experienced a refusal of healthcare or delay in treatment due to their HIV infection [14]. Nowadays, in the era when all patients with HIV infection ideally start treatment as soon as possible after diagnosis, and many patients are virologically suppressed, the risk of transmission has decreased even further, and while there are no targeted trials to show how low this risk really is, it is safe to assume that it is close to zero in a patient with undetectable viral load at the moment of performing the intervention [15]. Refusing to provide treatment to a patient is an unacceptable practice and only reinforces the vicious circle of stigma, followed by self-stigma and reluctance to disclose diagnosis, which may in turn lead to inadequate care or care that is not fully adapted to the patient’s medical needs [16].

Increasing year of study was significantly correlated with better knowledge of VPDs. This effect was also seen in a different questionnaire-based study from Iași, Romania, where higher year students more accurately recognized HBV as a VPD [17]. However, an important finding of our current study is that increasing year of study was not correlated with self-perceived risk and neither with the willingness to treat patients with chronic viral infections, which highlights an important unmet need and suggests that the current university curricula should be revised to include more targeted information regarding the actual dimension of the risk of transmission of HBV, HCV, and HIV in dental practice. 

Furthermore, only 2–3% of students accurately recognized that respecting the standard precautions and the current disinfection and sterilization regulations would indeed protect them and subsequent patients from the cross-transmission of infection [18]. This is clearly shown by the fact that over three quarters of the surveyed students stated that they would need to take supplementary precaution measures in case they treated a patient with a bloodborne viral infection. 

Almost half of our survey respondents had experienced some type of accidental occupational exposure during their relatively short clinical practice. Among these, most (55.0%) did not investigate the source patient’s bloodborne virus status and 80.5% did not consult an infectious disease specialist for guidance related to post-exposure prophylaxis (PEP). This is in line with the above-mentioned questionnaire-based study from Iași, which showed that only half (50.2%) of the students were aware of the key moments when testing for BBV should be performed after an accidental exposure [17]. In our current study, even among the few respondents who did consult an infectious disease practitioner, only a small fraction presented for the consult on the first day following exposure. These findings emphasize the need to increase knowledge about the steps that should promptly be taken by each practitioner to ensure appropriate post-exposure measures [15].

The lack of awareness regarding bloodborne viruses is also reflected in the very low rate of screening for viral infections, with more than half of those surveyed stating that they had never performed a test for HBV, HCV, or HIV. According to the 2023 updated recommendations from the US Centers for Disease Control and Prevention, everyone aged 18 years and older should be screened for HBV with a triple panel that includes HBsAg, anti-HBs, and total anti-HBc at least once during their lifetime and screening should be repeated periodically in those who remain at risk of acquiring infection [19]. We generally recommend yearly screening for HBV, HCV, and HIV, particularly for healthcare workers, and dental students should certainly be included in this recommendation [15]. However, students do not appear to be aware of the need to undergo screening, and this should be addressed through further educational activities.

We also identified issues that require improvement in the domain of understanding and implementing pre-exposure prophylaxis, with the rates of self-declared vaccination being incompatible with the clinical reality. For example, vaccination against HBV is included in the national immunization program (NIP) for newborns, followed by the completion of the primary vaccination regimen by the age of one year. The HBV vaccine coverage in Romania for the birth cohort 2000, corresponding to the median age of respondents, was 98% [20]. This suggests that the 49.5% self-reported rate of vaccination is more likely a reflection of the rate of students who were aware of or had access to medical records regarding their childhood vaccination status, rather than the real vaccine coverage. The same can be said for the diphtheria–tetanus–*pertussis* vaccine, which is administered in Romania through the NIP as primary series in the first year of life, followed by boosters at 6 and 14 years old, and for which the national coverage was 99% for the birth cohort 2000 [21]. However, the participants in this study self-reported the rate of vaccination for this vaccine at 67.3%. Furthermore, only 17% of them stated that they had received a tetanus–diphtheria-containing booster within the past 10 years, suggesting that this age group could indeed form a pool of non-immune individuals who are susceptible to important VPDs. This gap in adult booster vaccination could potentially be addressed by the newly amended immunization protocol in the country, updated in 2023 to now facilitate adult vaccination, but its implementation was not yet fully underway at the time when our survey was performed.

These self-reported vaccination rates are lower than what would be reasonably expected, which could potentially be indicative of the anti-vaccine movement [22,23,24], which has indeed decreased the vaccination rates in the county in the past years and has also influenced the perceptions of those who had already been vaccinated in the past. Important gaps in knowledge and awareness regarding VPDs have also been reported among students from Poland in an article that highlights the importance of implementing undergraduate-level educational activities on the topic of vaccination [23].

However, we have also identified self-reported rates of vaccination that are higher than those expected, i.e., 87.1% vaccination against COVID-19, which is much higher than the overall average vaccine coverage in the country [25]. However, higher vaccine willingness has been reported among medical students during the COVID-19 pandemic [26], and many of them actually experienced the COVID-19 pandemic during their studies, with important numbers of medical and dental students having volunteered to help during the initial pandemic waves [27,28], so this might be a true reflection of a higher vaccine acceptance rate in this student category, which could be an encouraging finding. 

The current study is limited by the relatively small sample size and by the representativity of the respondents particularly for one of the dental universities in the country, despite having been shared with three geographically different universities. With approximately 200 students per year and 6 years of study in total, the response rate remains below 10%. This low response rate could, in itself, be an indicator of the low interest that dental students have regarding notions such as IPC, VPDs, BBVs, and PEP, which are considered to be outside of their direct field of practice. This should be addressed through raising awareness, as low interest may drive low knowledge, which in turn was associated in our study with higher perceived risk and lower willingness to treat patients with BBV infections or to adhere to vaccine prophylaxis. The predominance of female respondents should not be considered a limitation of the current findings, as it reflects the overall trend in the country, where female students represent the large majority of dentistry students. The current study also has important strengths, i.e., providing an in-depth understanding of the correlation between the knowledge, perceptions, and attitudes of dental students regarding infectious diseases and infection prevention. 

Notably, this study has highlighted important areas where targeted practical education should further be implemented during dental school in order to increase, on the one hand, the safety of the dental practitioner (i.e., by the correct uptake of standard precautions and vaccine prophylaxis), and the equity and inclusivity of the delivered dental care. 

## 5. Conclusions

Among the surveyed dental students, we identified high awareness regarding correct PPE use, and practices were significantly correlated with the perceived usefulness of PPE. Better knowledge and awareness are needed regarding vaccine-preventable diseases, with a focus on understanding one’s vaccination history and the need for adult-age boosters. We also identified an important need to raise awareness regarding the importance of undergoing routine screening for bloodborne viruses among dental students. Furthermore, most survey respondents incorrectly considered themselves to be at high or very high risk of acquiring bloodborne viruses during routine dental practice. We propose an adaptation to the university curriculum to increase emphasis on the dental practitioner’s very low actual risk of acquiring bloodborne pathogens, such as HBV, HCV, or HIV, and on the existence and documented efficacy of prompt post-exposure measures in case of occupational exposure. Consequently, based on our current findings, we hypothesize that improving students’ knowledge and awareness would also translate into a higher willingness to treat patients with chronic viral infections and into a safer and more inclusive dental practice. These are key areas proposed for targeted focus to empower future practitioners and to facilitate the improvement of across-discipline metacompetences for infection prevention and control.

## Figures and Tables

**Table 1:** Characteristics of the survey respondents (*n* = 101).

Respondent Characteristics	Number (Percentage)
**Gender**	
Female	91 (90.1%)
Male	10 (9.9%)
**Year of study**	
1	3 (3.0%)
2	14 (13.9%)
3	23 (22.8%)
4	4 (4.0%)
5	12 (11.9%)
6	45 (44.6%)
**Involvement in clinical activities**	
Yes	80 (79.2%)
No	19 (18.8%)
Prefer not to respond	2 (2.0%)

**Table 2:** Perceived risk, attitudes, and practices related to working with patients with chronic viral infections (n = 101). HBV, hepatitis B virus; HCV, hepatitis C virus; HIV, human immunodeficiency virus.

Respondent Characteristics	HBV Infection	HCV Infection	HIV Infection
**Self-perceived risk**			
Low	13 (12.9%)	14 (13.9%)	15 (14.9%)
Medium	21 (20.8%)	22 (21.8%)	24 (23.8%)
High	30 (29.7%)	32 (31.7%)	29 (28.7%)
Very high	37 (36.6%)	33 (32.7%)	33 (32.7%)
**Attitude towards future procedures**			
Refuse to treat	3 (3.0%)	5 (5.0%)	11 (10.9%)
Perform only minimal-bleeding procedures	42 (41.6%)	42 (41.6%)	39 (38.6%)
Perform any procedures, including surgery	56 (55.4%)	54 (53.5%)	51 (50.5%)
**Correlation: perceived risk vs. attitude**	τ_b_ = −0.214, *p* = 0.018	τ_b_ = −0.234, *p* = 0.013	τ_b_ = −0.242, *p* = 0.006
**Supplementary precautionary measures**			
No, this is not necessary	2 (2.0%)	3 (3.0%)	2 (2.0%)
Yes, for myself	90 (89.1%)	89 (88.1%)	89 (88.1%)
Yes, for sterilizing the dental instruments	95 (94.1%)	94 (93.1%)	96 (95.0%)
Yes, for disinfecting the dental unit	76 (75.2%)	76 (75.2%)	76 (75.2%)

## Data Availability

The data that support the findings of this study are available from the corresponding author upon reasonable request.
